# Supplementary material for: The Use of Mobile Health Interventions for Outcomes among Middle-Aged and Elderly Patients with Prediabetes: A Systematic Review
Source: Int J Environ Res Public Health. 2022 Oct 20;19(20):13638. doi: 10.3390/ijerph192013638 (PMC9603799; doi:10.3390/ijerph192013638)
Supplement: Supplementary file 1 [file ijerph-19-13638-s001.zip › supplementary File S2 Screening Forms.pdf]

Additional File S2. Screening forms

Title and abstract screening

1. Does this reference discuss the use of mobile health interventions for outcomes among middle-aged and elderly patients with prediabetes?

    ❍ **Yes/unclear***

    ❍ No

*Those answered yes/unclear will be passed through to full-text screening.

Full-text screening

1. Language of publication

    ❍ **English**

    ❍ Other ______________________

2. Does this article a:

    RCTs, quasi-experimental trials, non - randomized controlled studies (cohort study, case-control), non - randomized studies without a reference group (e.g., cohort study, cross-sectional), or case series (minimum 5 cases)?

    ❍ **Yes**

    ❍ No (select one of the options: case series with less than five cases or other publication type (Editorials, opinions, literature reviews, conference abstracts and proceedings, protocols, suggestions,

consensus, qualitative research, and grey literature, such as reports, theses, and annotations))

❍ Abstract

3. Does the population include adult (18 years and above) participants who had prediabetic state with criteria according to American Diabetes Association (ADA) or World Health Organization (WHO) without history of T2DM and diabetes medications?

   ❍ Yes

   ❍ No

   ❍ Mixed population (prediabetic and diabetic state)

   ❍ Unclear (contact authors)

4. [If answer to question 3 is mixed population] If this article includes a mixed population, do they provide prediabetic and diabetic state specific outcomes data?

   ❍ Yes

   ❍ No

Typically, these questions are nested. If an answer allows us to proceed in the inclusion criteria, the next question will appear. Those bolded would be those that would pass through to the following question.
