# Supplementary material for: The Use of Mobile Health Interventions for Outcomes among Middle-Aged and Elderly Patients with Prediabetes: A Systematic Review
Source: Int J Environ Res Public Health. 2022 Oct 20;19(20):13638. doi: 10.3390/ijerph192013638 (PMC9603799; doi:10.3390/ijerph192013638)
Supplement: Supplementary file 1 [file ijerph-19-13638-s001.zip › supplementary File S3 Data Extraction Forms.pdf]

Additional File S3. Data Extraction Forms

Review title or ID	The Use of Mobile Health Interventions for Outcomes Among Middle-Aged and Elderly Patients with Prediabetes : Protocol of Systematic Review	
Study ID		
Report ID		
Notes		
Date form completed *(dd/mm/yyyy)*		
Name/ID of person collecting data		
Reference citation		
Study author contact details		
Source of funding		
Study funding source (including role of funders)		
Possible conflicts of interest (for study authors)		
Notes		
DO NOT PROCEED IF STUDY EXCLUDED FROM REVIEW		
Study Characteristic		
Study setting		
Study design		
Study duration		
Inclusion/exclusion criteria		
Type of Mobile Health Application/Interventions;		
Number of centers (if applicable)		
Sample size		
Population Characteristics		
	Intervention	Comparator
Age		
Race/Ethnicity,		
Socioeconomic Status (E.G., Income, Level Of Education),		
Other Risk Factors For Prediabetic State,		
Body Measurement Status,		

Blood Glucose Level		
Other		
Outcomes		
	Intervention	Comparator
Number Needed To Treat		
Odds Ratio		
Relative Ratio		
Risk Difference		
Applicability		
Have important populations been excluded from the study? (consider disadvantaged populations, and possible differences in the intervention effect)		
Is the intervention likely to be aimed at disadvantaged groups? (e.g. lower socioeconomic groups)		
Does the study directly address the review question? (any issues of partial or indirect applicability)		
Notes:		
